# Supplementary material for: Enhanced clinical assessment of hematologic malignancies through routine paired tumor and normal sequencing
Source: Nat Commun. 2023 Oct 28;14:6895. doi: 10.1038/s41467-023-42585-9 (PMC10613284; doi:10.1038/s41467-023-42585-9)
Supplement: Supplementary file 1 — Supplementary Information [file 41467_2023_42585_MOESM1_ESM.pdf]

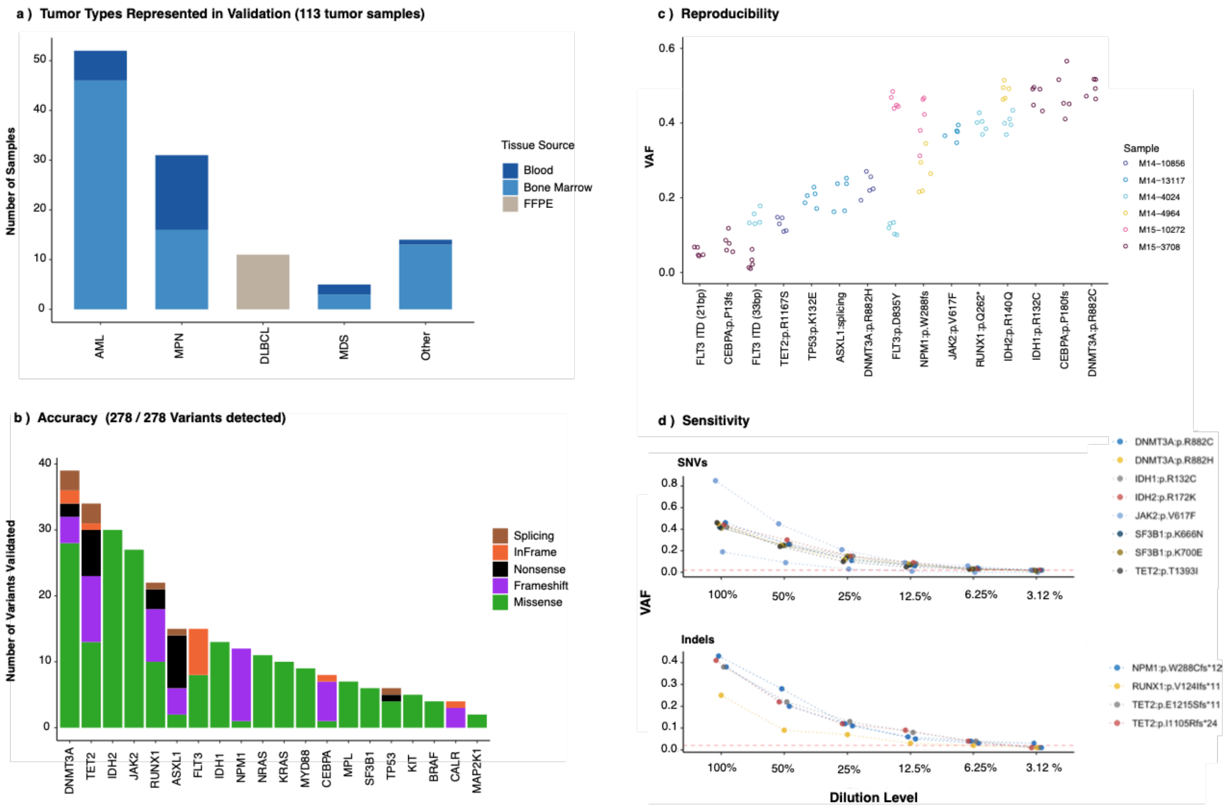

Supplementary Figure 1: MSK-IMPACT Heme validation study. a)Tumor and tissue types sequenced for MSK-IMPACT Heme assay validation. b) accuracy, c) reproducibility, and d) sensitivity results. Source data are provided as a Source Data file.

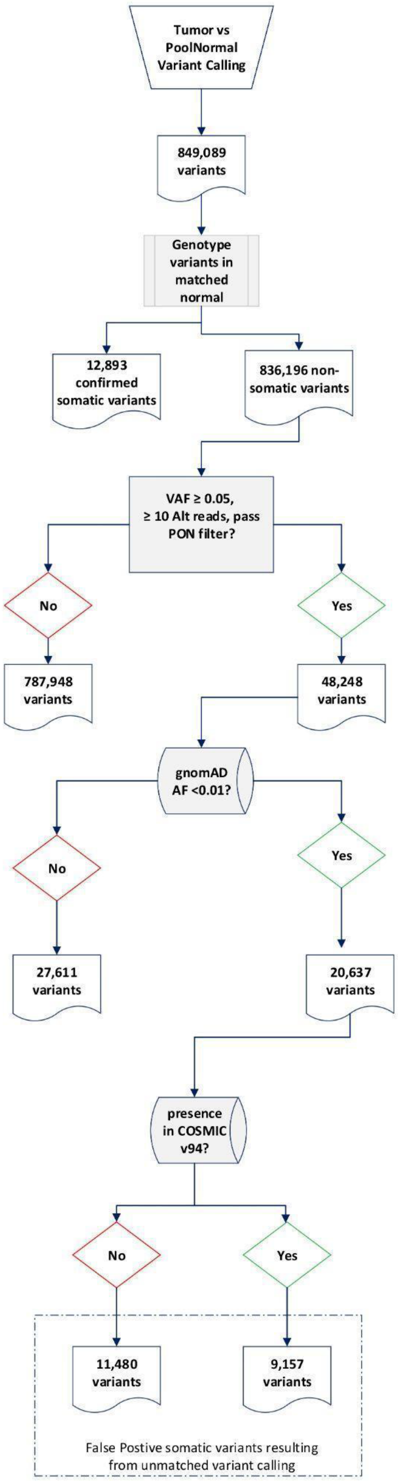

Supplementary Figure 2: Schematic of tumor-only variant calling analysis process and results using a threshold of 1% MAF in the gnomAD database.

a)

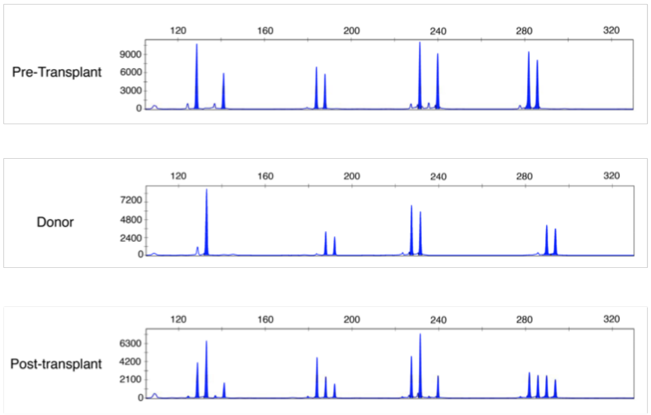

b)

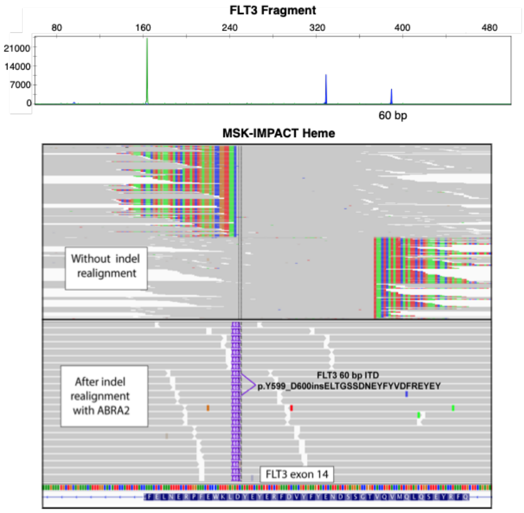

Supplementary Figure 3: Example patient case highlighting ability of FACETs2n to detect complex mutations in a post-transplant chimeric patient. (a) STR analysis of pre- and post-transplant patient bone marrow and donor blood samples (b) FLT3 fragment analysis (top) and indel realignment with MSK-IMPACT Heme are concordant for identification of a 60bp ITD.

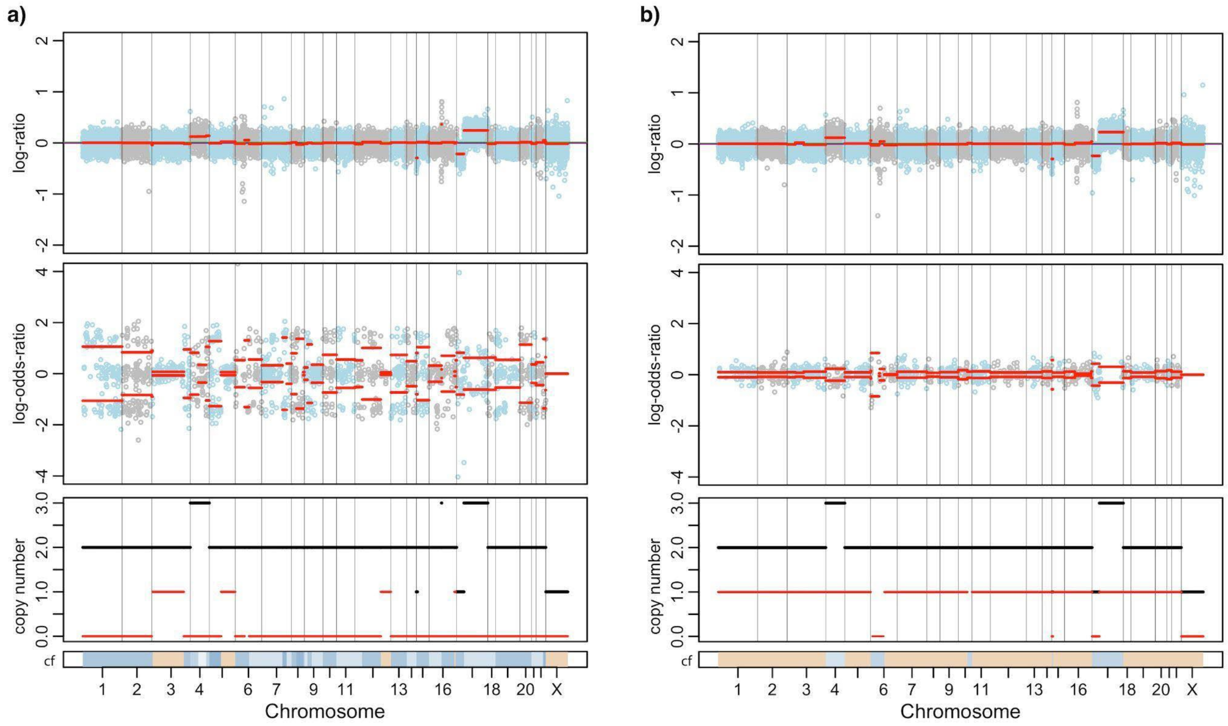

Supplementary Figure 4: Genome wide allele-specific copy number profile of a post-transplant patient obtained using (a) the baseline host germline reference and (b) the intersection of heterozygous SNPs between baseline host and baseline donor germline reference samples. Using a baseline reference from only one individual results in the inability to accurately identify regions of allelic imbalance due to host-donor chimerism in the post-transplant setting.

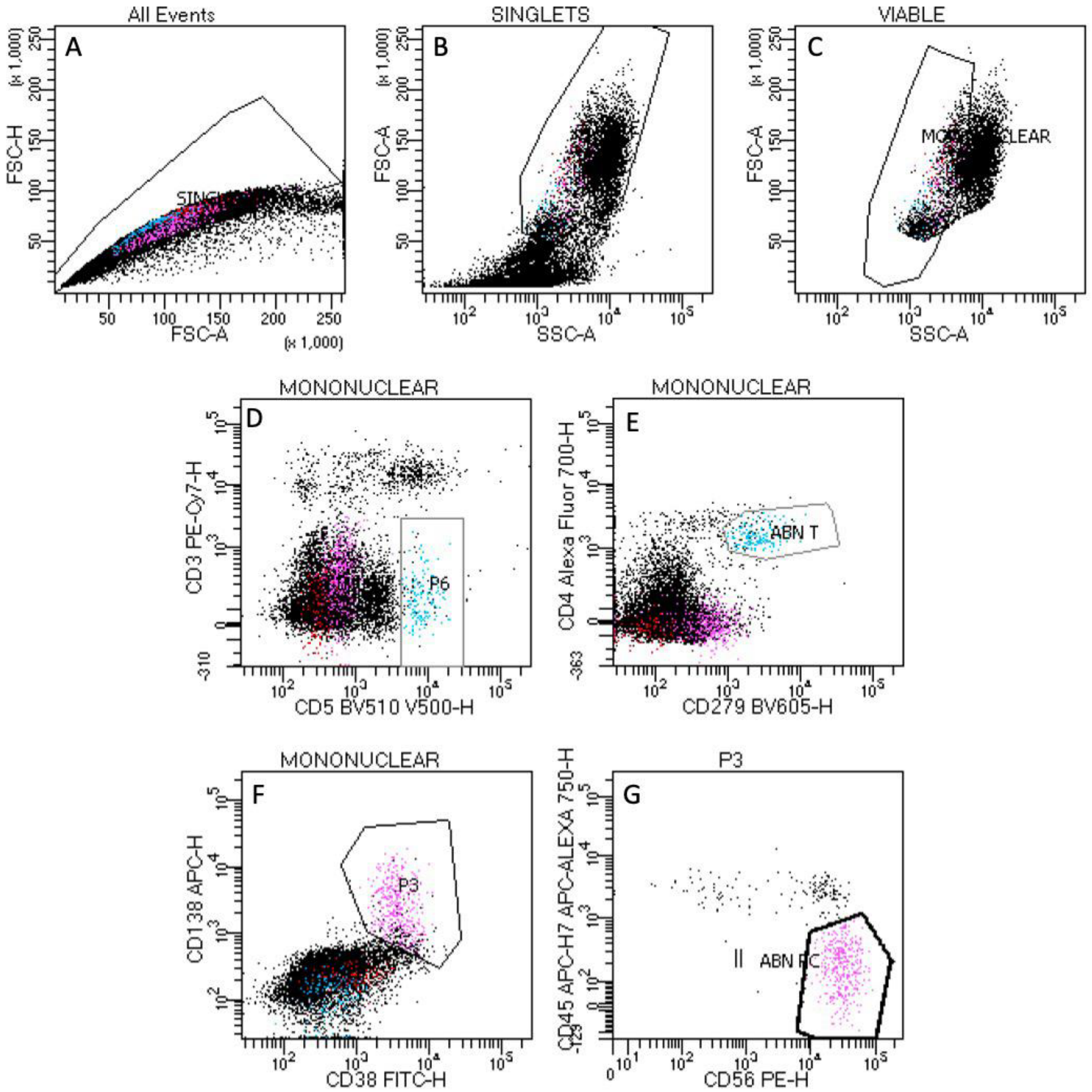

Supplementary Figure 5: Flow cytometry gating and sorting strategy for enrichment of specific cell populations. The plots demonstrate gating strategies to enrich for A) singlet events, B) viable cells, C) viable mononuclear cells, D) CD3-negative, CD-5 positive cells, E) abnormal T-cells with expression of CD4 and CD279, F) plasma cells with expression of CD38 (bright) and CD138, and G) abnormal plasma cells with aberrant expression of CD56.

Supplementary Table 1: MSK-IMPACT Heme gene list

ABL1	BRD4	DTX1	GNAS	KDM6A	NF1	RAD21	SMO
ACTG1	BRIP1	DUSP22	GNB1	KDR	NF2	RAD50	SOCS1
AKT1	BTG1	EED	GRIN2A	KEAP1	NFE2	RAD51	SOX2
AKT2	BTK	EGFR	GSK3B	KIT	NFE2L2	RAD51B	SP140
AKT3	CALR	EGR1	H1-2	KMT2A	NKX2-1	RAD51C	SPEN
ALK	CARD11	EP300	H2BC5	KMT2B	NOTCH1	RAD51D	SPOP
ALOX12B	CASP8	EP400	H3C2	KMT2C	NOTCH2	RAD52	SRC
AMER1	CBFB	EPHA3	H3C8	KMT2D	NOTCH3	RAD54L	SRSF2
APC	CBL	EPHA5	HDAC1	KMT5A	NOTCH4	RAF1	STAG1
AR	CCND1	EPHA7	HDAC4	KRAS	NPM1	RARA	STAG2
ARAF	CCND2	EPHB1	HDAC7	KSR2	NRAS	RB1	STAT3
ARHGEF28	CCND3	ERBB2	HGF	LCK	NSD1	REL	STAT5A
ARID1A	CCNE1	ERBB3	HIF1A	LMO1	NT5C2	RET	STAT5B
ARID1B	CD274	ERBB4	HIST1H1B	LTB	NTRK1	RHOA	STAT6
ARID2	CD28	ERG	HIST1H1D	MALT1	NTRK2	RICTOR	STK11
ARID3A	CD58	ESCO2	HIST1H1E	MAP2K1	NTRK3	RNF43	SUFU
ARID3B	CD79A	ESR1	HIST1H2AC	MAP2K2	P2RY8	ROBO1	SUZ12
ARID3C	CD79B	ETNK1	HIST1H2AG	MAP2K4	PAK7	ROS1	SYK
ARID4A	CDC73	ETV6	HIST1H2AL	MAP3K1	PALB2	RPTOR	TBL1XR1
ARID4B	CDH1	EZH2	HIST1H2AM	MAP3K13	PARP1	RRAGC	TBX3
ARID5A	CDK12	FANCA	HIST1H2BC	MAP3K14	PAX5	RTEL1	TENT5C
ARID5B	CDK4	FANCC	HIST1H2BG	MAPK1	PBRM1	RUNX1	TERT
ASXL1	CDK6	FANCD2	HIST1H2BJ	MAPK3	PCBP1	RUNX1T1	TET1
ASXL2	CDK8	FAS	HIST1H2BK	MCL1	PDCD1	SAMHD1	TET2
ATM	CDKN1B	FAT1	HIST1H2BO	MDM2	PDGFRA	SDHA	TET3
ATP6AP1	CDKN2A	FBXO11	HLA-A	MDM4	PDGFRB	SDHB	TGFBR2
ATP6V1B2	CDKN2B	FBXW7	HNF1A	MED12	PDPK1	SDHC	TNFAIP3
ATR	CDKN2C	FGF19	HRAS	MEF2B	PDS5B	SDHD	TNFRSF14
ATRX	CEBPA	FGF3	ID3	MEN1	PHF6	SETBP1	TOP1
ATXN2	CHEK1	FGF4	IDH1	MET	PIGA	SETD1A	TP53
AURKA	CHEK2	FGFR1	IDH2	MGA	PIK3C2G	SETD1B	TP53
AURKB	CIC	FGFR2	IGF1	MGAM	PIK3C3	SETD2	TP63
AXIN1	CIITA	FGFR3	IGF1R	MITF	PIK3CA	SETD3	TRAF2
AXL	CRBN	FGFR4	IGF2	MLH1	PIK3CG	SETD4	TRAF3
B2M	CREBBP	FLCN	IKBKE	MOB3B	PIK3R1	SETD5	TRAF5
BACH2	CRKL	FLT1	IKZF1	MPEG1	PIK3R2	SETD6	TSC1
BAP1	CRLF2	FLT3	IKZF3	MPL	PIM1	SETD7	TSC2
BARD1	CSF1R	FLT4	IL7R	MRE11	PLCG1	SETDB1	TSHR
BCL10	CSF3R	FOXL2	INPP4B	MSH2	PLCG2	SETDB2	TYK2
BCL11B	CTCF	FOXO1	IRF1	MSH6	PMS2	SF3B1	U2AF1
BCL2	CTNNB1	FOXP1	IRF4	MTOR	PNRC1	SGK1	U2AF2
BCL6	CUX1	FURIN	IRF8	MUTYH	POT1	SH2B3	UBR5
BCOR	CXCR4	FYN	IRS2	MYC	PPP2R1A	SMAD2	VAV1
BCORL1	CYLD	GATA1	JAK1	MYCL	PRDM1	SMAD4	VAV2
BCR	DAXX	GATA2	JAK2	MYCN	PRKAR1A	SMARCA4	VHL
BIRC3	DDR2	GATA3	JAK3	MYD88	PTCH1	SMARCB1	WHSC1
BLM	DDX3X	GNA11	JARID2	NBN	PTEN	SMARCD1	WT1
BRAF	DIS3	GNA12	JUN	NCOR1	PTPN1	SMC1A	XBP1
BRCA1	DNMT3A	GNA13	KDM5A	NCOR2	PTPN11	SMC3	XPO1
BRCA2	DOT1L	GNAQ	KDM5C	NCSTN	PTPN2	SMG1	ZRSR2

Supplementary Table 2: Effect of gnomAD MAF filtering threshhold on number of reportable and potentially clinical actionable variants

gnomAD MAF Threshold	# Non-somatic variants <gnomAD threshhold	# Non-somatic variants <gnomAD threshhold that would change ICC/WHO classification*	% Cases with Non-somatic variant that would result in change to ICC/WHO classification*	# Non-somatic variants <gnomAD threshhold in COSMIC v94	Average # COSMIC variants <gnomAD threshhold per sample	% Cases with a COSMIC variant <gnomAD threshhold
1%	20,637	42	1.80%	9,157	4	95.30%
0.50%	15,946	42	1.80%	5,958	2.8	89.50%
0.10%	10,523	42	1.80%	3,121	2	68.50%

*Determination of whether a variant would alter ICC/WHO classification was determined by interrogating variant calls in the following genes: *ASXL1, BCOR, CEBPA, EZH2, IKZF1, NPM1, PAX5, RUNX1, SF3B1, SRSF2, STAG2, TP53, U2AF1, ZEB2,* and *ZRSR2.*

Supplementary Table 3: Germline variants in *DNMT3A*, *TET2*, and *TP53* filtered through use of matched normal

Gene	VariantClass	AAchange	Normal_VAF	Tumor_VAF	OncoTree Code
DNMT3A	Missense_Mutation	p.N501S	0.473	0.51001	MDS Workup
DNMT3A	Missense_Mutation	p.R55H	0.403	0.50403	PMF
DNMT3A	Missense_Mutation	p.E152K	0.444	0.49054	PMF
DNMT3A	Missense_Mutation	p.L639V	0.466	0.49645	TAML
TET2	Missense_Mutation	p.P174H	0.47	0.48516	AML
TET2	Missense_Mutation	p.P1655L	0.389	0.4856	AML
TET2	Missense_Mutation	p.V1718L	0.489	0.45425	AML
TET2	Missense_Mutation	p.R814C	0.48	0.47845	AML
TET2	Missense_Mutation	p.P1575L	0.419	0.49209	AML
TET2	Missense_Mutation	p.R814C	0.42	0.52903	AML
TET2	Missense_Mutation	p.H800Y	0.483	0.48808	AML
TET2	Missense_Mutation	p.R1572Q	0.469	0.54439	AML
TET2	Missense_Mutation	p.H800Y	0.456	0.51434	AML
TET2	Missense_Mutation	p.H800Y	0.488	0.48103	AML
TET2	Missense_Mutation	p.H800Y	0.459	0.45229	AML
TET2	Missense_Mutation	p.R1498P	0.487	0.4661	AML
TET2	Missense_Mutation	p.R1498P	0.468	0.46892	AML
TET2	Missense_Mutation	p.P1575L	0.419	0.49819	AML
TET2	Missense_Mutation	p.P1575L	0.419	0.50332	AML
TET2	Missense_Mutation	p.G1519R	0.417	0.48665	AML
TET2	Missense_Mutation	p.P174H	0.435	0.34118	AML
TET2	Missense_Mutation	p.R1465Q	0.443	0.49271	CHIP
TET2	Missense_Mutation	p.P174H	0.435	0.50529	CML
TET2	Missense_Mutation	p.R1095K	0.517	0.40264	CMML
TET2	Missense_Mutation	p.Y1589C	0.483	0.44882	CMML
TET2	Missense_Mutation	p.S1775N	0.405	0.46604	ET
TET2	Missense_Mutation	p.P1962L	0.525	0.43248	HDCN
TET2	Missense_Mutation	p.N275K	0.458	0.52224	HDCN
TET2	Missense_Mutation	p.G453S	0.426	0.44207	HDCN
TET2	Missense_Mutation	p.G453S	0.426	0.48491	HDCN
TET2	Missense_Mutation	p.R581H	0.492	0.42308	HDCN
TET2	Missense_Mutation	p.R814C	0.471	0.45896	MDS
TET2	Missense_Mutation	p.R1498P	0.468	0.42275	MDS
TET2	Missense_Mutation	p.N767D	0.51	0.46517	MDS
TET2	Missense_Mutation	p.R1498P	0.471	0.47845	MDS
TET2	Missense_Mutation	p.R1095K	0.481	0.48071	MDS
TET2	Missense_Mutation	p.E1010D	0.491	0.49574	MDS
TET2	Missense_Mutation	p.N1743D	0.496	0.48712	MDS
TET2	Missense_Mutation	p.V1718L	0.482	0.45598	MDS
TET2	Missense_Mutation	p.G429R	0.392	0.49561	MDS
TET2	Missense_Mutation	p.Q810R	0.47	0.48105	MDS Workup
TET2	Missense_Mutation	p.Q1068K	0.503	0.46073	MDS Workup
TET2	Missense_Mutation	p.S145N	0.381	0.48674	MDS Workup
TET2	Missense_Mutation	p.Q810R	0.461	0.51684	MDS Workup
TET2	Missense_Mutation	p.Y192H	0.502	0.49441	MNM
TET2	Missense_Mutation	p.H1036Y	0.483	0.48139	MPN
TET2	Missense_Mutation	p.R1993L	0.46	0.47385	MPN
TET2	Missense_Mutation	p.R1993L	0.46	0.51145	MPN
TET2	Missense_Mutation	p.R814C	0.463	0.5	MPN Workup
TET2	Missense_Mutation	p.N275K	0.528	0.48084	MPN Workup
TET2	Missense_Mutation	p.V1426M	0.418	0.48571	PMF
TP53	Missense_Mutation	p.Q317K	0.457	0.44129	AML
TP53	Missense_Mutation	p.P4L	0.452	0.48894	CHIP
TP53	Missense_Mutation	p.V31I	0.403	0.4258	MPN

Supplementary Table 4: SCNA validation accuracy

	SNP-Array CNVs present	SNP-Array CNVs not present	Total
**(MSK-IMPACT)**	130	0	130
**(MSK-IMPACT)**	10	756	766
**Total**	140	756	896

Supplementary Table 5: SCNA validation sensitivity study for 12p Gain

Sample ID	Dilution (%)	Arm	Tumor	Total copy number	Lower copy number	cf.em. impact	Results by MSK-IMPACT Heme
M19-29320	100%	12p gain	DLBCL	18	1	89%	GAIN
M19-29320-50	50%	12p gain	DLBCL	19	1	44%	GAIN
M19-29320-25	25%	12p gain	DLBCL	22	1	21%	GAIN
M19-29320-12	12.00%	12p gain	DLBCL	9	1	<20%	GAIN
M19-29320-6	6.00%	12p gain	DLBCL	5	1	<20%	GAIN

Supplementary Table 6: SCNA validation sensitivity study for 13q loss

Sample ID	Dilution (%)	Arm	Tumor	Total copy number	Lower copy number	cf.em. impact	Results by MSK-IMPACT Heme
M19-29320	100%	13q loss	DLBCL	1	0	89%	LOSS
M19-29320-50	50%	13q loss	DLBCL	1	0	44%	LOSS
M19-29320-25	25%	13q loss	DLBCL	2	0	21%	LOSS
M19-29320-12	12.00%	13q loss	DLBCL	1	0	<20%	LOSS
M19-29320-6	6.00%	13q loss	DLBCL	2	1	100%	DIPLOID
