# Supplementary material for: Enhanced clinical assessment of hematologic malignancies through routine paired tumor and normal sequencing
Source: Nat Commun. 2023 Oct 28;14:6895. doi: 10.1038/s41467-023-42585-9 (PMC10613284; doi:10.1038/s41467-023-42585-9)
Supplement: Supplementary file 3 — Reporting Summary [file 41467_2023_42585_MOESM3_ESM.pdf]

nature portfolio

Corresponding author(s): Mark D. Ewalt, Ahmet Zehir, Maria E. Arcila

Last updated by author(s): Sep 19, 2023

Reporting Summary

Nature Portfolio wishes to improve the reproducibility of the work that we publish. This form provides structure for consistency and transparency in reporting. For further information on Nature Portfolio policies, see our Editorial Policies and the Editorial Policy Checklist.

Statistics

For all statistical analyses, confirm that the following items are present in the figure legend, table legend, main text, or Methods section.

n/a	Confirmed	
☐	☒	The exact sample size (*n*) for each experimental group/condition, given as a discrete number and unit of measurement
☐	☒	A statement on whether measurements were taken from distinct samples or whether the same sample was measured repeatedly
☐	☒	The statistical test(s) used AND whether they are one- or two-sided<br>*Only common tests should be described solely by name; describe more complex techniques in the Methods section.*
☒	☐	A description of all covariates tested
☒	☐	A description of any assumptions or corrections, such as tests of normality and adjustment for multiple comparisons
☐	☒	A full description of the statistical parameters including central tendency (e.g. means) or other basic estimates (e.g. regression coefficient) AND variation (e.g. standard deviation) or associated estimates of uncertainty (e.g. confidence intervals)
☐	☒	For null hypothesis testing, the test statistic (e.g. *F*, *t*, *r*) with confidence intervals, effect sizes, degrees of freedom and *P* value noted<br>*Give P values as exact values whenever suitable.*
☒	☐	For Bayesian analysis, information on the choice of priors and Markov chain Monte Carlo settings
☒	☐	For hierarchical and complex designs, identification of the appropriate level for tests and full reporting of outcomes
☒	☐	Estimates of effect sizes (e.g. Cohen's *d*, Pearson's *r*), indicating how they were calculated

Our web collection on statistics for biologists contains articles on many of the points above.

Software and code

Policy information about availability of computer code

Data collection	No software was used for data collection.
Data analysis	R version 4.0.1 was used to analyze the data in this study; R code used is available on GitHub. The following packages were used in our analyses. Sequencing data for the IMPACT study was analyzed using BWA (version 0.7.5a), ABRA (version 0.92), ABRA2 (version 2.13), Genome Analysis Toolkit(GATK) (version 3.3-0), Mutect (version 1.1.4), VarDict (version 1.4.6), Somatic Indel Detector (version 2.3), VEP (version 86), FACETS2n (v0.3.0)

For manuscripts utilizing custom algorithms or software that are central to the research but not yet described in published literature, software must be made available to editors and reviewers. We strongly encourage code deposition in a community repository (e.g. GitHub). See the Nature Portfolio guidelines for submitting code & software for further information.

Data

Policy information about availability of data

All manuscripts must include a data availability statement. This statement should provide the following information, where applicable:

- Accession codes, unique identifiers, or web links for publicly available datasets
- A description of any restrictions on data availability
- For clinical datasets or third party data, please ensure that the statement adheres to our policy

The raw sequencing data for the MSK-IMPACT analysis is protected and cannot be broadly available due to privacy laws; patient consent to deposit raw sequencing

data was not obtained. All results derived from the analysis of clinical sequencing data (mutations, copy number alterations, and structural variants) are publicly available. Analysis of germline alterations was not performed due to lack of patient consent for dedicated germline analysis and reporting. The minimal clinical and somatic alteration data (including mutations and allele specific copy number calls) necessary to replicate the findings in the article are publicly available on cBioPortal: https://www.cbioportal.org/study?id=heme_msk_impact_2022 and have been deposited to https://github.com/mskcc/MSK_IMPACT_HEME. Source data are provided with this paper.

Research involving human participants, their data, or biological material

Policy information about studies with human participants or human data. See also policy information about sex, gender (identity/presentation), and sexual orientation and race, ethnicity and racism.

Reporting on sex and gender	We have utilized sex information for patients involved in the study, however, gender information was not collected.
Reporting on race, ethnicity, or other socially relevant groupings	We have not collected race, ethnicity, or other socially relevant groupings of the patients as they were not relevant to the analyses.
Population characteristics	We have not collected population characteristics of the patients as they were not relevant to the analyses.
Recruitment	There was no specific recruitment utilized. Patients in this study are seen by MSK oncologists, prospectively, and their samples were sequenced accordingly. This may have potentially led to biases based on the population of patients at MSK (specific diagnoses and/or socioeconomic factors).
Ethics oversight	The study was approved by MSKCC Institutional Review and Privacy Board.

Note that full information on the approval of the study protocol must also be provided in the manuscript.

Field-specific reporting

Please select the one below that is the best fit for your research. If you are not sure, read the appropriate sections before making your selection.

☒ Life sciences ☐ Behavioural & social sciences ☐ Ecological, evolutionary & environmental sciences

For a reference copy of the document with all sections, see nature.com/documents/nr-reporting-summary-flat.pdf

Life sciences study design

All studies must disclose on these points even when the disclosure is negative.

Sample size	Tumor genomic data from 1,937 patients whose tumor samples were sequenced using MSK-IMPACT Heme were used. We did not perform sample size calculations as all patients sequenced were included in this study. This samples sized was deemed sufficient, as this is the largest individual center dataset of matched hematologic oncology patients reported to date and the availability of this data will support additional studies in the field.
Data exclusions	No samples were excluded.
Replication	Each patient's sample was sequenced only once and no replication was performed. Replication was not required as we utilized a validated clinical assay and these tests are not reported when providing information for patient management and therefore were deemed sufficiently accurate (as detailed in our validation studies) to not require replication.
Randomization	Randomization was not relevant as there was no intervention associated with this study and all patients included had their tumor samples sequenced on MSK-IMPACT Heme.
Blinding	Sample and patient identifies were de-identified before analysis.

Reporting for specific materials, systems and methods

We require information from authors about some types of materials, experimental systems and methods used in many studies. Here, indicate whether each material, system or method listed is relevant to your study. If you are not sure if a list item applies to your research, read the appropriate section before selecting a response.

Materials & experimental systems

n/a	Involved in the study
☒	☐ Antibodies
☒	☐ Eukaryotic cell lines
☒	☐ Palaeontology and archaeology
☒	☐ Animals and other organisms
☐	☒ Clinical data
☒	☐ Dual use research of concern
☒	☐ Plants

Methods

n/a	Involved in the study
☒	☐ ChIP-seq
☒	☐ Flow cytometry
☒	☐ MRI-based neuroimaging

Clinical data

Policy information about clinical studies

All manuscripts should comply with the ICMJE guidelines for publication of clinical research and a completed CONSORT checklist must be included with all submissions.

Clinical trial registration: NCT01775072

Study protocol: https://clinicaltrials.gov/study/NCT01775072

Data collection: Samples were collected from patients enrolled in this trial who had IMPACT-Heme testing on one of their samples. These samples included in-patient and out-patient collections of peripheral blood, bone marrow, lymph node, and other biopsy specimens.

Outcomes: Genomic profiling results were reported back to the ordering clinician and the patient through clinical reports. This is a retrospective study and the only endpoint is the result of the clinical test.
